# Feasibility study on the use of single-port laparoscopic surgery for diagnosis and tumor sampling in advanced epithelial ovarian cancer — Case series of three cases

**DOI:** 10.1016/j.ijscr.2023.108212

**Published:** 2023-04-26

**Authors:** Akihiko Misawa, Eizo Kimura, Kazuki Asai, Aiko Oka, Kiyono Osanai, Atsushi Suzuki

**Affiliations:** Department of Obstetrics and Gynecology, Kosei Hospital, 2-25-1 Wada, Suginami-ku, Tokyo 166-0012, Japan

**Keywords:** Advanced epithelial ovarian cancer (AEOC), Laparoscopic surgery, Single-laparoscopic surgery (SLPS), Predictive index (PI) scoring

## Abstract

**Introduction:**

In advanced epithelial ovarian cancer (AEOC), it is often difficult to achieve optimal surgery at primary debulking surgery (PDS) due to intra-abdominal dissemination and/or metastasis. When it is determined that optimal surgery is not possible, neoadjuvant chemotherapy (NAC) is performed prior to subsequent debulking surgery. Also, a histological diagnosis of the tumor is very important before initiation of NAC. Laparoscopic surgery is thus useful to objectively diagnose whether an optimal primary debulking surgery is feasible and to obtain tumor biopsy samples. In order to minimize the invasive procedures at initial surgery, we performed laparoscopic surgery using a single-port method.

**Case presentation:**

Three patients were diagnosed as stage IV ovarian cancer based on imaging and physical examination. Single-port laparoscopic surgery was performed. The intraabdominal findings were evaluated in all patients by predictive index scoring and objectively diagnosed as not ideal candidates for optimal surgery at PDS. Our use of single-port laparoscopic surgery (SPLS) allowed for safe surgical outcomes and sufficient tissue sampling for histological diagnosis.

**Clinical discussion:**

Laparoscopic surgery is not appropriate for tumor reduction surgery in AEOC; however, its use as an alternative method to laparotomy is recommended for tumor tissue biopsy and/or intraperitoneal observation. Previous studies have reported on the use of conventional multi-port laparoscopic surgery. The single-port method, when compared to conventional laparoscopic surgery, is less invasive with only one abdominal wound at the umbilicus.

**Conclusion:**

SPLS is feasible and clinically useful for diagnosis and tumor sampling in AEOC.

## Highlights

• Optimal tumor reduction is the surgical objective in advanced ovarian cancer.
• Preoperative chemotherapy is recommended for suboptimal cases at primary surgery.
• In preoperative chemotherapy, tumor sampling is necessary for histopathological diagnosis.
• Single-port laparoscopic surgery is a feasible and clinically useful method for diagnosis and tumor sampling.

## 1 Introduction

In AEOC, optimal surgery (less than 1 cm in maximum diameter of residual tumor) or complete surgery (no residual tumor) is the surgical objective. However preoperative chemotherapy (neoadjuvant chemotherapy; NAC) plus interval debulking surgery (IDS) is recommended for cases in which optimal surgery is not possible at PDS [1]. Extensive intra-peritoneal tumor spread is often observed with tumor extension not only to the lymph nodes, but also to the entire abdominal cavity including the retroperitoneum, the retroperitoneal meshwork, the mesentery, and the diaphragm into the thoracic cavity thus rendering optimal surgery impossible at PDS. In such cases, as tumor sampling is necessary for histological diagnosis, in lieu of biopsy of the main tumor, a readily biopsied lymph node or other tissue mass, or cell blocks derived from either ascites or pleural effusions have been used prior to the introduction of laparoscopic surgery. Similarly, the decision whether optimal or complete surgery is feasible at initial surgery would be based on imaging diagnosis or by abdominal findings at laparotomy. Laparoscopic surgery is not appropriate for tumor reduction surgery in AEOC; however, its use as an alternative method to laparotomy is recommended for tumor tissue biopsy and/or intraperitoneal observation as a minimally invasive method [1], [2]. The predictive index (PI) is useful for evaluating whether optimal tumor reduction is feasible. The PI is an objective score based on abdominal findings including omental cake, peritoneal carcinomatosis, diaphragmic carcinomatosis, mesenteric retraction, bowel infiltration, stomach infiltration, and liver metastases [3].

We performed an initial feasibility study using SPLS as a minimally invasive method in three AEOC cases.

This article has been reported in line with PROCESS criteria [4].

## 2 Presentation of case

### 2.1 Case study

We initiated a feasibility study in January 2019 to investigate the use of SPLS for the diagnosis and tumor sampling of AEOC cases in which imaging studies indicate that optimal surgery was not possible at initial surgery. We conducted this study because we believe that SPLS for AEOC is beneficial as it is 1) less invasive due to the size of the small wound size (2.5 cm), 2) less invasive due to one port placement, 3) possible to initiate early anticancer drug treatment postsurgery, and 4) possible to reduce the number of cancer port implantation sites due to fewer ports.

## 3 Results

Three patients with suspected stage IV ovarian cancer based on preoperative MRI and CT imaging were assessed in this study. Imaging diagnosis demonstrated ovarian cancer with peritoneal dissemination, lymph node metastasis, distant metastases and it was determined that PDS was not possible. SPLS was performed to observe the intraabdominal cavity and evaluate tumor spread using PI scoring. A tumor biopsy was also performed for histopathological diagnosis.

**Fig. 1:**
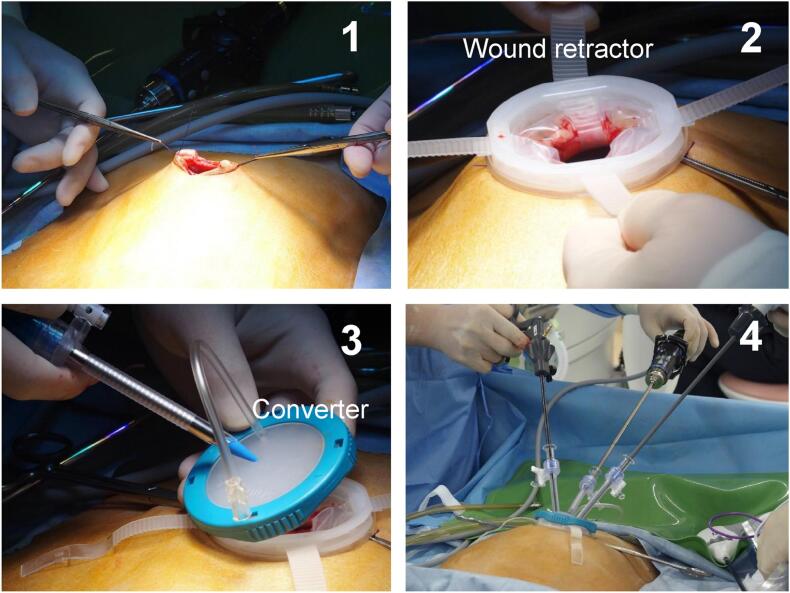
Single-port laparoscopic surgery using X-gate free. 1. A 2.5 cm incision is made at the umbilicus. 2. The X-gate free wound retractor is applied to the wound, and the flaps are pulled outward. 3. The converter is placed on the wound retractor, and trocars are inserted. 4. Laparoscopic surgery is started.

All patients underwent SPLS with a 2.5 cm incision at the umbilicus. An X-gate free wound retractor (Sumitomo Bakelite Co., Ltd., Tokyo) and three EZ Access ports (Hakko, Tokyo) were used as the single port. The laparoscopic visualization system was a 5-mm flexible scope (Olympus Corporation, Tokyo) (Fig. 1).

Case 1 is a 43-year-old woman (gravida 0 and parturition 0) with regular menstruation cycles with no previous medical or family history. The patient initially consulted with her physician due to irregular bleeding and a 4-month history of lower abdominal pain. A pelvic mass was discovered, and she was referred to our institution for treatment. Lower gastrointestinal endoscopy revealed a mass in the sigmoid colon identified by biopsy as adenocarcinoma of gynecological origin. Stage IVB was suspected.

Case 2 is a 63-year-old woman (gravida 1 and parturition 0) with a prior history of a total hysterectomy for uterine fibroids at 25 years of age and with no family history. The patient was referred to our hospital with lower abdominal pain. A pelvic mass and lymph node metastases which were demonstrated especially in the supraclavicular and mediastinum regions were found on CT scan. Stage IB was suspected.

**Fig. 2:**
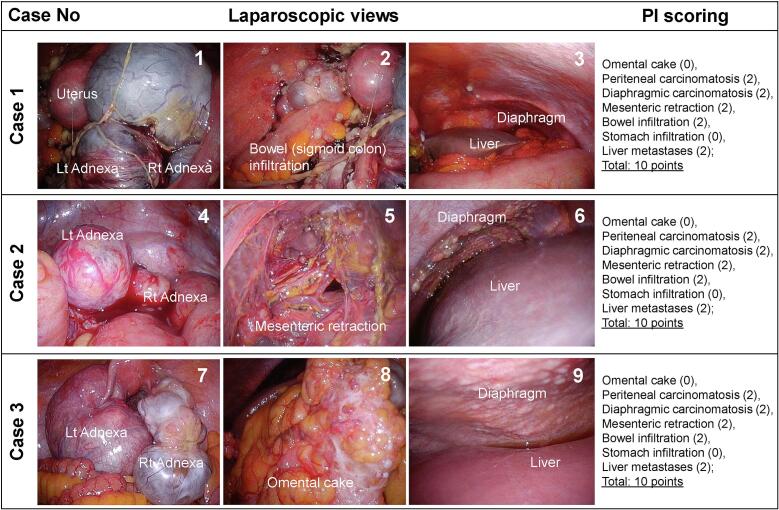
Laparoscopic views and PI scoring in case. 1 Uterus and adnexa; 2. bowel (sigmoid colon) infiltration; 3. diaphragmic carcinomatosis and liver metastases. 4. Adnexa; 5. mesenteric retraction; 6. diaphragmic carcinomatosis and liver metastases. 7. Uterus and adnexa; 8. omental cake; 9. diaphragm and liver metastases. PI score. Case 1: Omental cake (0), peritoneal carcinomatosis (2), diaphragmic carcinomatosis (2), mesenteric retraction (2), bowel infiltration (2), stomach infiltration (0), liver metastases (2). Total: 10 points. Case 2: Omental cake (0), peritoneal carcinomatosis (2), diaphragmic carcinomatosis (2), mesenteric retraction (2), bowel infiltration (2), stomach infiltration (0), liver metastases (2). Total: 10 points. Case 3: Omental cake (0), peritoneal carcinomatosis (2), diaphragm carcinomatosis (2), mesenteric retraction (2), bowel infiltration (2), stomach infiltration (0), liver metastases (2). Total: 10 points.

Case 3 is a 60-year-old woman (gravida 5 and parturition 4) with menopause at 50 years and a history of type 2 diabetes mellitus but with no family history. The patient's initial visit to our institution was for emergency admission due to abdominal pain. Pleural effusions were found, from which the cytological diagnosis was adenocarcinoma. Stage IVB was suspected (Fig. 2).

All cases underwent SPLS under general anesthesia. In all cases, extensive peritoneal dissemination was observed and the predictive index was 10 points. Optimal debulking surgery was determined to not be feasible and tumor biopsy was performed for histopathological diagnosis. Operative time was less than 60 min, and patients were discharged from the hospital on the fourth postoperative day without intraoperative or postoperative complications. All cases revealed high-grade serous carcinoma. The post-discharge course was uneventful, and all cases started postoperative cyclic chemotherapy within 1 to 3 weeks. After three courses of TC chemotherapy (paclitaxel 180 mg/m^2^, carboplatin AUC 6 every 3 weeks), all cases were confirmed to have PR on imaging, and IDS was performed. Complete surgery was possible in all cases. The use of SPLS improved patient QOL and also allowed early diagnosis of the tumor and timely initiation of treatment; patient satisfaction was improved (Table 1).

**Table 1:** Clinical characteristics of the patients.

	Case 1	Case 2	Case 3
Age	43	63	60
Preoperative stage	Stage IVB	Stage IVB	Stage IVA
Predictive index	10	10	10
Laparoscopic method	Tumor sampling (peritoneal dissemination)	RSO + pOMT + tumor sampling (peritoneal dissemination)	RSO + tumor sampling (peritoneal dissemination)
Operation time (min)	56	49	59
Pathological diagnosis	HGSC	HGSC	HGSC
Bleeding	little	little	little
Initiation of chemotherapy after laparoscopy (days)	7	19	6
NAC	TC	TC	TC
IDS	Complete surgery	Complete surgery	Complete surgery
Surgical method	TAH + BSO + PLN + PAN + OMT + sigmoid colon resection + appendectomy	LSO + PLN + PAN + OMT + diaphragm and Douglas peritoneal stripping	TAH + LSO + OMT + PLN + PAN
Chemotherapy effect	Moderate	Moderate	Moderate
Postoperative stage	ypT3c pN0 pM1	ypT3c, pN1, pM1	ypT3c, N1, M1a

TAH: total abdominal hysterectomy, RSO: right salpingo-oophorectomy, BSO: bilateral salpingo-oophorectomy, LSO; left salpingo-oophorectomy, PLN; pelvic lymphadenectomy, PAN; para-aortic lymphadenectomy, (p)OMT; (partial) omentectomy.

## 4 Discussion

The surgical goal for AEOC is maximum tumor debulking, including peritoneal dissemination and metastatic lesions. Optimal surgery, indicating a residual tumor of 1 cm or less, has been shown to improve disease prognosis, while complete surgery, indicating complete surgical removal of the tumor, is associated with an even better disease prognosis [5], [6], [7], [8]. However, in AEOC, optimal PDS is achieved in 38 %–40 % of cases, while complete surgery is reported in only 12 %–19 % of cases [6], [7], [8]. On the other hand, in NAC followed by IDS, optimal surgery is reported in 18 %–38 % of cases, while complete surgery is reported in 39 %–64 % of cases [6], [7], [8]. In clinical hindsight, it can be assumed that most cases of suboptimal surgeries at PDS may have benefited from NAC prior to interval debulking surgery instead of suboptimal PDS followed by chemotherapy.

Determining whether optimal or complete surgery can be achieved is a key clinical decision often based upon imaging diagnosis or a surgeon-based decision depending on intra-peritoneal findings at initial laparotomy, but there is no standard evaluation system. However, it has been shown that the peritoneal cancer index (PCI), based upon spread of abdominal dissemination, is correlated with disease prognosis but no correlation has been demonstrated with level of maximal debulking at surgery. Diagnostic laparoscopic surgery coupled with a PI evaluation has also been shown to be a highly reliable diagnostic method [9]. It has been shown that patients with a PI score of 8 (4 parameters positive) or higher are not candidates for PDS. In cases with a PI score of 8 or higher, it is predicted that patients would have difficulty achieving optimal surgery.

In all t cases, optimal surgery at PDS was not possible because the PI was 10 points at SPLS. Most importantly, following NAC, complete IDS was achieved in all cases.

A comparison of SPLS versus laparotomy in the surgical staging of early ovarian cancer has shown that laparoscopic surgery using a single-port access method is a viable surgical option. [10] However, there have been few published reports on SPLS for AEOC; reports published to date have studied the possibility of intra-abdominal observation, the PCI scaring and tumor biopsy but have not evaluated the surgical outcomes [11], [12]. Our results demonstrate that SPLS could be feasible in the intraabdominal evaluation and tumor sampling of AEOC cases.

Our institution has routinely performed SPLS via the umbilical approach for benign gynecologic diseases. PI evaluation was possible in all three of our cases using SPLS and it was also possible to obtain sufficient tissue samples for pathological diagnosis. From a pathological standpoint, based on consultation with our resident pathologist during the establishment of the surgical protocol for this study, we aimed to 1) resect several tumor samples of 1 cm^2^ or greater so that the heat necrosis of tissue from energy devices would not affect the pathological diagnosis, and 2) resect tissue from the primary tumor, if technically possible, to allow more accurate diagnosis. These techniques allowed sufficient sampling of viable tumor tissue. Recent advances in treatment strategies have required tumor tissue in sufficient quantity to allow for possible drug selection and/or molecular testing. This approach is especially necessary in cases of NAC failure, as it may be important to determine the second and subsequent courses of chemotherapy treatment based on tumor profiles. In all our cases, we performed the SPLS successfully and obtained sufficient tumor biopsy in a relatively short operative time with minimal blood loss. It is also important to note that we did not experience any surgical complications. Our results showed the benefits we believed. Based on these findings, we conclude that the SPLS is a safe and feasible option for AEOC diagnostic laparoscopy.

## 5 Conclusion

SPLS for intraperitoneal observation and tissue collection avoids invasive laparotomy. SPLS is a viable option for laparoscopic review of AEOC.

## Informed consent

Written informed consent was obtained from the patient for publication of this case study and accompanying images. A copy of the written consent is available for review by the Editor-in-Chief of this journal on request.

## Ethical approval

This study is exempt from ethical approval at our institution.

## Funding

Not applicable.

## Guarantor

Akihiko Misawa.

## Research registration number

Not applicable.

## CRediT authorship contribution statement

A.M.: Writing – original draft, Conceptualization. E.K.: Writing – review & editing, Supervision. K. A.: Investigation, Data curation. A. O.: Investigation, Data curation, Writing – original draft. K. O.: Literature review, Chart review. A. S.: Writing - review & editing.

## Declaration of competing interest

The authors declare that they have no known competing financial interests or personal relationships that could influence the work reported in this paper.

## Data Availability

All data generated or analyzed during this study are included in this published article.
